# Geographic delay characterization of railway systems

**DOI:** 10.1038/s41598-021-00361-z

**Published:** 2021-10-21

**Authors:** Mark M. Dekker

**Affiliations:** 1 Department of Information and Computing Sciences, Utrecht University, Princetonplein 5, 3584 CC Utrecht, The Netherlands; 2 Centre for Complex Systems Studies, Utrecht University, Minnaertgebouw, Leuvenlaan 4, 3584 CE Utrecht, The Netherlands

**Keywords:** Civil engineering, Complex networks

## Abstract

Railway systems provide pivotal support to modern societies, making their efficiency and robustness important to ensure. However, these systems are susceptible to disruptions and delays, leading to accumulating economic damage. The large spatial scale of delay spreading typically make it difficult to distinguish which regions will ultimately affected from an initial disruption, creating uncertainty for risk assessment. In this paper, we identify geographical structures that reflect how delay spreads through railway networks. We do so by proposing a graph-based, hybrid schedule and empirical-based model for delay propagation and apply spectral clustering. We apply the model to four European railway systems: the Netherlands, Germany, Switzerland and Italy. We characterize these geographical delay structures in the railway systems of these countries and interpret these regions in terms of delay severity and how dynamically disconnected they are from the rest. The method also allows us to point out important differences between these countries’ railway systems. For practitioners, such geographical characterization of railways provides natural boundaries for local decision-making structures and risk assessment.

## Introduction

Transport systems provide a core function to our society, moving passengers and goods around the globe to allow for global trade, business and leisure. Efficient infrastructure and mobility has been found important for economies to grow^1^. However, at severe economic costs, the efficiency of many such systems is regularly affected by perturbations and subsequent spread of disruptions. Examples can be found in trade networks being affected by epidemics^2^, earthquakes^3^ or cyclones^4^, energy and internet problems due to power outages^5^, railway disruptions due to natural hazards^6–9^ and many more. The economic costs of such events, also on smaller scales, motivate scholars and practitioners to investigate the robustness and resilience of these systems to perturbations^10–12^, and to understand and predict subsequent evolution of perturbations^13,14^. This paper focuses on understanding the structure of the evolution of such perturbations in a subset of transport systems: railway systems.

Railway systems involve the on-time geographical transport of passengers or goods, utilizing resources (*assets*, like physical trains or crew) according to a predefined schedule, from, via and towards nodes (e.g., stations) in a network, along certain routes or tracks (edges). While the on-time dynamics are described in a pre-defined schedule, of interest here are the temporal deviations from the schedule, which are referred to as *delays*, calculated as the executed time minus the predefined time—in situations with no delay at all, all resources run on time and no delay is present; hence, no perturbation or associated dynamics is present. What sets apart railway systems from many other transport systems, is their dependence on a detailed pre-determined system, in some countries even down to the particular assets used per activity^14^. This attribute, in combination with usually high utilization of existing capacity commonly causes perturbations that are an interesting (and necessary) topic of study. In the remainder of this paper, when we talk about ‘dynamics’ of railway systems, we actually refer to the spreading and change of *delays* rather than the scheduled movement of the assets—note that the latter is usually a well-optimized plan that contains, by construction, little interacting elements (although the timetable itself may be prone to cascading-like phenomena that in turn affect the delay dynamics^14^).

The research on transport delays can be split into multiple parts. A first part focuses on understanding the origin of initial perturbations, involving research on natural disasters as mentioned above, but also on smaller-scale fluctuations of activity delays that may give rise to systemic delay generation^6,15–17^. Oftentimes, these studies are case-specific, due to the heterogeneous (and often external) nature of such initial perturbations. Another line of transport delay research focuses on prediction of delays: the simulation of how a currently delayed situation will evolve to a (future) situation. Many kinds of approaches to this problem exist: micro simulation tools including a high level of detail on each edge^18,19^, larger-scale stochastic and analytic models both on the planning and realization side^13,16,20–22^, and in more recent years, machine learning or data-driven studies are contributing to the field^7,23–25^. A third branch of research involves the understanding of delay dynamics on a more structural level: characterizing the nature and (semi-)universal ‘laws’ without the specific aim of prediction. For example, finding macroscopic nation-wide patterns in railway delay^7^, characterizing mechanisms of how trains pass delay onto other assets^21^, how the interaction between crew, rolling stock and line planning can lead to delay cascades^14^.

This paper contributes to the third branch of transport (or railway) delay literature and focuses on the large spatial scale that is usually associated with these systems. We aim to identify geographic regions that act as subsystems, partially independent in terms of delays, and these clusters are reinterpreted in terms of their role in the global system, which add to the understanding of the general structure of these often nation-wide systems. The identification of such regions contributes to the overall understanding of the dynamics of delays, providing answers to questions like ‘where is delay generally generated, propagated through and attracted?’ and ‘what regions are dynamically near-isolated and may be treated as such?’. These questions are useful for practitioners to not only help in prediction methods, but also for more strategic decisions on infrastructure planning and updates of future timetables.

Identifying substructures in complex systems such as railways is commonly done using clustering algorithms, with applications ranging from physics to ecology^26^, climate^27^ and even epidemics^28^. The wide variety of applications comes with a multitude of clustering methodologies. Graph-based clustering is often based on random-walks and modularity-optimization principles^29^, like the famous Louvain clustering method^30^, or on spectral properties of core graph matrices^31^. A famous data-based clustering method is *K*-means^32^, while many other methods exist. It is important to note the difference between identifying substructures in the network topology alone, and doing so by accounting for spatial structures in dynamic processes happening on top of the network topology^33^. In this paper, we focus on the latter, using spectral clustering. The main reason for choosing spectral clustering as opposed to any modularity-optimization tool is that spectral modes and the shape of the eigenspectrum reveal more than just the detection of communities (as we see later in Fig. 3 of this paper). But indeed, there are important advantages of modularity optimization, as well, such as the automatic optimization of the amount of detected clusters.

Also in transportation literature, clustering techniques have been applied, e.g., to assist real-time management, operations and decision making^34–37^. Other papers apply clustering tools on statistical variables to identify general states in the system^7,38,39^. Even though compartmentalizing the geographical system based on observed or simulated delay patterns is less common, it is not new, e.g. concerning the identification of communities in cargo ships^40^, assessing topological properties in the Swiss railway network^41^ or quantifying resilience in the Indian railways^8^. However, combining data on the dynamics of the (delay of the) system, with data on the infrastructure has rarely been done in railway literature. This, and the subsequent interpretation of those found communities, is the goal of this paper. It is important to emphasize that mere (topological) clustering of only the infrastructure, neglecting all other dynamical and operational information, would provide clusters that have less of an operational meaning: delay does not diffuse equally along directions that are topologically equivalent. The relation between more general diffusion dynamics among subregions in a networks has been investigated theoretically before^42^, but to our knowledge, this has never been applied to characterize dynamic structures in railway systems.

The paper is structured as follows. The model and the associated clustering method is discussed and applied to a fictitious transport network in “Methods”. In “Application to European railway systems”, we apply the methods to real data of four European railway systems and show the results of the model and clustering. We interpret the clusters in terms of connectivity and role in the country’s delay in “Interpretation and relation to daily operations”. We end with several conclusive remarks in “Conclusion”.

## Methods

In this section, we build a graph-based model for delay dynamics based on infrastructure and empirical train delays. We start by separating the underlying infrastructure from the delay dynamics that happens on top. The underlying infrastructure, consisting of *nodes*, being stations or departure/arrival locations, and *edges* as the railway tracks between them, is assumed to remain invariant in this analysis—different from, for example network perturbation analyses or node-failure transport problems^5,8,43^. We henceforth use the term ‘nodes’ and ‘stations’ interchangeably. The aim of this paper is to provide insights in the geographic aspects of delay propagation. To this end, we distinguish factors that result in spatial non-uniformity: e.g., edges with a higher frequency of trains, fewer parallel tracks and re-routing options, are more prone to propagate or amplify delay than other edges. Rather than purely looking at the topology of the underlying infrastructure network, it is such non-uniformity that defines weights of the edges that largely impact the resulting spectral clustering. We note that there are also other types of non-uniformity in railway systems that are not directly related to geography that are outside of the scope of this paper.

We distinguish two types of spatial non-uniformities: (1) those consequential to traffic flows, as found in the timetable, and (2) those that are due to other effects, which we infer from delay statistics. Traffic flows are static properties that affect delay propagation and can be determined from the system’s timetable, e.g., (planned) running times and resource travel frequency. These factors are used to imply what portion of existing delay at a station is propagated in each possible direction. Spatial non-uniformities unrelated to the timetable, in contrast, relate to dynamic factors affecting delays and are derived from delay statistics rather than from the static (non-delayed) timetables. These factors act as multipliers: if, from the timetable, we expect delay to be transported in a certain direction, we use statistics to estimate whether it amplifies or dampens. Below, we build a model where we define both of these factors.

### Spatial non-uniformity in traffic flows

**Figure 1:**
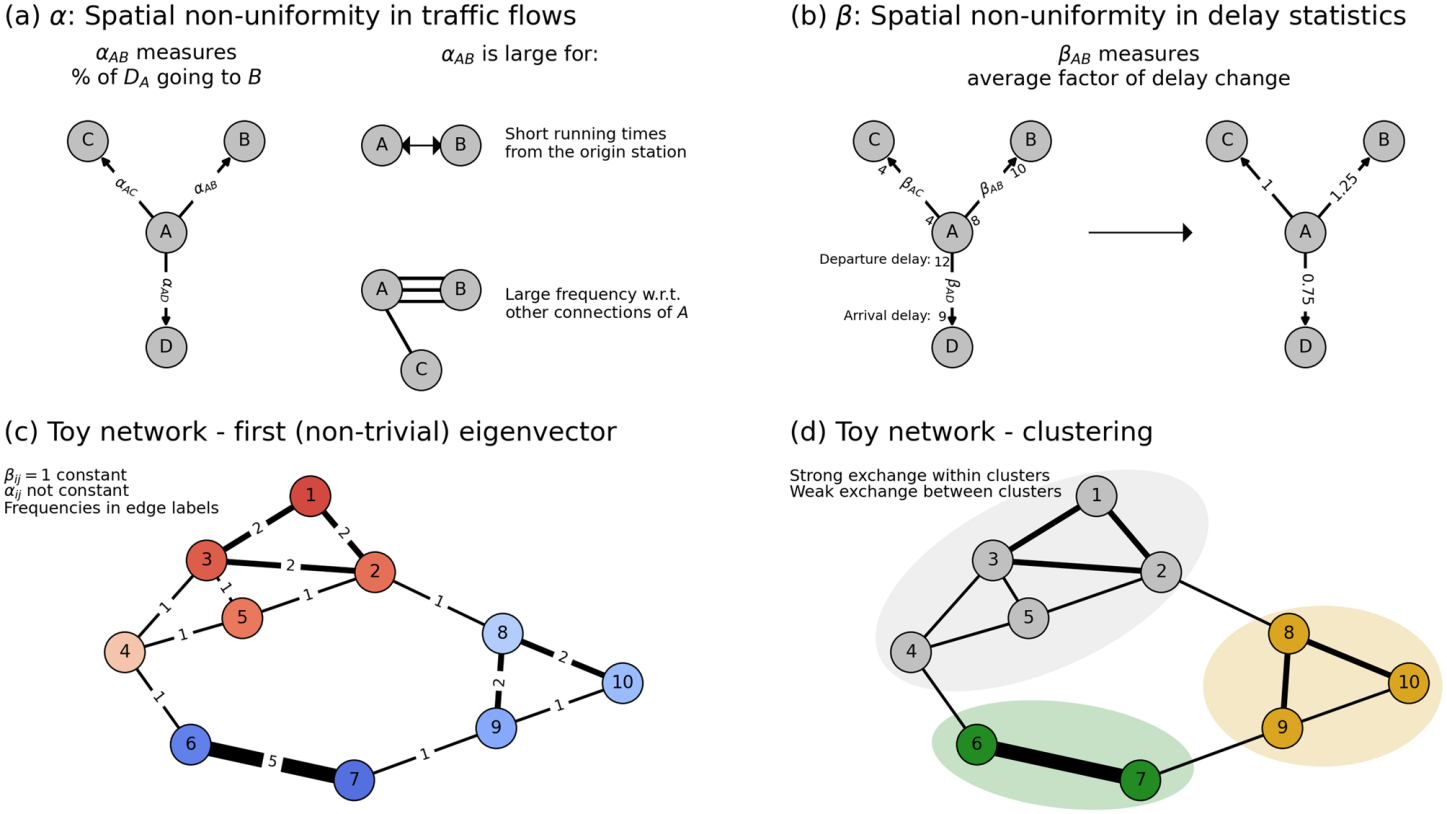
(**a**) Illustration of the spatial non-uniformity in traffic flows $\alpha$. Note that $\alpha _{ij}$ also contains a term concerning the running time of trains from *i*, but the proportions of all outgoing links from *i* remain in tact; hence the description of $\alpha _{ij}$ as the fraction of delay in *i* going to *j*. (**b**) Illustration of the spatial non-uniformity in delay statistics $\beta$. (**c**) First non-trivial eigenvector of an example network where we assumed $\beta$ constant, and $\alpha$ only variant because of changing transport frequencies (denoted in edge width and labels). Eigenvector coefficients are shown in node colors, from blue to red. (**d**) Clustering of the example network, where $K=3$

Spatial non-uniformity in traffic flows comes from within the system’s internal predefined properties, like the timetable. The intuition is that edges with more traffic propagate delay more easily and busier routes involve more congestion. In particular, delays are carried along with trains in the direction of their trajectories and nodes are more easily affected by a neighboring node’s delays if the link between them contains high-frequent traffic, a mechanism that is commonly used in delay propagation models^21,22^. We capture the spatial non-uniformity in traffic flows by identifying the proportional direction of delay propagation from any node towards each of its edges, using the relative frequencies *f* of trains. Another spatial non-uniformity in the timetable concerns the fact that in some areas, the edges are very short, leading to short running times $\tau$. Combining these two factors of spatial non-uniformity in traffic flows results in a single edge weighing factor $\alpha$ (see Fig. 1a) for each edge *ij* between nodes *i* and *j* (i.e., $i, j \in \{ 1, \ldots , N\}$ where *N* is the amount of of stations, and *i* and *j* make a direct connection in the railway network):

1 $$\begin{aligned} \alpha _{ij} = \frac{f_{ij}\cdot \tau _{\text {min}}}{\sum _{j'}f_{ij'} \cdot \tau _{i}}, \end{aligned}$$

where $\tau _{\text {min}}$ is the system-wide minimum running time. The factor $\tau _{\text {min}}/\tau _i$ therefore reflects changes in the weights of $\alpha$ of all edges of node *i* based on its running times relative to the minimum.

### Spatial non-uniformity due to other effects

Besides spatial non-uniformity in traffic flows, there are also factors outside from the timetable that increase or decrease delays, depending on the area in the railway network. Examples of such factors are a large amount of block signals, fewer parallel tracks, speed limits, high volume of passengers (delaying boarding times), decreased vision and increased chances of infrastructure problems or trees falling on tracks. In our model, we derive these factors in an aggregated way from data by comparing delays of trains before and after crossing every edge. Specifically, we determine the edge weighing factor $\beta _{ij}$ for such spatial non-uniformities for each edge *ij* as follows:

2 $$\begin{aligned} \beta _{ij} = \frac{\langle D_{arr}^{ij} \rangle }{\langle D_{dep}^{ij}\rangle }, \end{aligned}$$

where $\langle D_{dep}^{ij}\rangle$ and $\langle D_{arr}^{ij}\rangle$ denote the observed average departure and arrival delays of trains moving from node *i* to *j* (i.e., across edge *ij*), respectively. In other words, it quantifies how much delay changes along this edge: $\beta _{ij} <1$ indicates that delays are, on average, decreased, while $\beta _{ij} >1$ indicates an average increase of delays when passing through edge *ij* [We take the averages in both the numerator and the denominator in Eq. (2) to prevent near-zero arrival or departure delay times from strongly altering the $\beta$ value of the edge]. Examples and the intuition of $\beta$ are illustrated in Fig. 1b.

### Model

The multiplications of the spatial non-uniformity factors $\alpha$ and $\beta$ result in weights of edges that reflect how delay geographically spreads through a railway network. This can be viewed as a delay propagation model, although we never use it to actually simulate delays—we use it to identify communities. The intuition of this model is as follows. Consider two stations I and II, connected by edge *ij*. Given an initial delay of $D_0$ at station I, it can be deduced from the timetable that a fraction $\alpha _{ij}$ of $D_0$ is propagated towards II—the rest, $(1-\alpha _{ij})\cdot D_0$, either remains at I or travels towards other stations. During the propagation of $\alpha _{ij} \cdot D_0$ towards II, the delay is increased or decreased on edge *ij* by a factor $\beta _{ij}$. In other words, the delay arriving at II in the next time step is $\alpha _{ij} \beta _{ij} D_0$. We generalize this reasoning by constructing the matrix *M* by element-wise multiplication of the factors $\alpha$ and $\beta$ for any pair of nodes *i* and *j* in the railway network:

3 $$\begin{aligned} M_{ij} = {\left\{ \begin{array}{ll} \alpha _{ij} \beta _{ij} &{}\text { if }i, j\hbox { are directly connected}\\ 1 - \sum _k \alpha _{ik} \beta _{ik} &{}\text { if }i=j\\ 0 &{} \text { elsewhere}, \end{array}\right. } \end{aligned}$$

As we are mainly interested in the dynamics and direction of delay propagation, rather than the absolute values of delay, we assume our model to conserve delay. This requires the matrix *M* to become row-stochastic, which is realized by defining the diagonal elements as in Eq. (3) to be $1 - \sum _k \alpha _{ik} \beta _{ik}$. We refer to the matrix *M* as the *dynamical* matrix, which, given a delay vector $\vec {D}(t)$ at time *t*, gives the delay vector at the next time step $t+\Delta t$ through the following multiplication:

4 $$\begin{aligned} \vec {D}(t+\Delta t) = \vec {D}(t) + \Delta t \cdot M \cdot \vec {D}(t), \end{aligned}$$

where $\Delta t$ refers to a time step, which is arbitrary and not of concern for the research in this paper. Both $\alpha$ and $\beta$ are dimensionless and we only use *M* to find out *structural* properties of the railway systems rather than for explicit delay modeling—i.e., even though Eq. (4) illustrates an interpretation of *M*, we do not use this equation.

### Properties of *M*

Many important model properties can be derived analytically from the row-stochastic matrix *M*, and we focus our research to the properties of this matrix. (Note that *M* contains averaged, time invariant, entries through the definitions of $\alpha$ and $\beta$ and therefore does not depend on time). Any delay simulation done using *M* eventually ends up with a ‘trivial’ delay vector with equal entries: $\vec {D}_i(t) = \vec {c}$ at all nodes *i*, where $\vec {c}_i := c = \frac{\vec {D}(t_0)}{N}$ with *N* the total amount of nodes. The trivial vector reflects that, given enough time, delay is spread in all corners of the graph (assuming it being connected). The question of how delay spreads in the *transient* is of interest to us, i.e., before this trivial solution. One way of identifying structural properties of a graph-based model is to look at the spectral modes. The intuition behind this is to find geographical delay patterns $\mathscr {D}$ that are persistent, i.e. slow to diffuse. Such patterns reveal natural boundaries that delays might not easily cross and geographical divisions into regions where delay is easily exchanged. In “Clustering”, we discuss how we use these spectral modes to define clusters. Mathematically, the problem of finding spectral modes $\mathscr {D}$ is defined in the eigenproblem of matrix *M*:

5 $$\begin{aligned} M\cdot \mathscr {D} = \lambda \mathscr {D} \end{aligned}$$

with eigenvectors $\mathscr {D}$ and eigenvalues $\lambda$. The closer $\lambda$ is to 1, the slower the delay decay in a (relative) delay distribution fixed by $\mathscr {D}$ [this follows directly from Eq. (5)]—i.e., the more persistent is the geographical delay pattern $\mathscr {D}$. In particular, there is one solution with $\lambda = 1$ such that $M\cdot \mathscr {D} = 1\cdot \mathscr {D}$, because *M* is row-stochastic. This is the aforementioned solution with constant coefficients, and corresponds to the first eigenvector. This does not provide any insight in the dynamic structure of the system, and is referred to as the ‘trivial’ eigenvector. The attribute of these eigenvectors being persistent, points to dynamical connections among nodes with approximately equal coefficients in an eigenvector. In other words, the coefficients of the eigenvectors can be used to find clusters of nodes that are have a dynamical connection, as we follow up on in the next section.

### Clustering

The clustering is based on the coefficients of the eigenvectors. The defining property of eigenvectors of *M* with eigenvalues close to 1 is that their coefficients are approximately conserved under multiplication with the matrix *M*, which through Eq. (4) defines the change of any delay vector. In other words, geographic delay distributions following the distribution of coefficients in an eigenvector are amplified over time. As illustrated in Fig. 1c, it therefore makes sense to cluster eigenvector coefficients based on their value. A single eigenvector provides interesting information already, which is why we propose a spectral approach in this paper. Combining multiple eigenvectors, however, allows us to do the actual clustering. Let us assume that we are searching for *K* clusters (the question of choosing *K* is addressed later). A common method of finding these clusters is by creating a *K*-dimensional embedding based on the first $K-1$ non-trivial eigenvectors (as the first eigenvector is constant and does not add any information), and applying a *K*-means algorithm to this space^26,31,44,45^. Indeed, this results in *K* clusters that are based on the respective differences between eigenvector coefficients. There are many more clustering methods, with various advantages and disadvantages. The advantage of *K*-means clustering is that it is one of the most well-known and intuitive Euclidean distance-based clustering methods. The disadvantage of *K*-means is that many implementations of the algorithm are not deterministic, and that *K* is not automatically defined.

We derive an appropriate value of *K* based on the so-called ‘eigengap heuristic’^31^, which is based on the eigenvalue spectrum. High (near-1) eigenvalues correspond to relatively persistent—and thus to us important—eigenvectors $\mathscr {d}$. Sudden ‘jumps’ in the eigenspectrum therefore point to a group of more important eigenvectors (those with higher $\lambda$) and the rest of the spectrum (those with lower $\lambda$) and can thus be used to distinguish which eigenvectors are therefore of interest. Assuming an equal amount of clusters one can distinguish with this set of eigenvectors (although the trivial eigenvector is not useful in this analysis), the largest ‘eigengap’ in the eigenspectrum defines *K*. Having a maximum amount of desired clusters (in the remained of this paper, we use $K_{max}=15$), results in:

6 $$\begin{aligned} K = \text {max} _{i=2} ^{15} \{\lambda _{i-1}-\lambda _{i}\}. \end{aligned}$$

Summarized: we start with the eigendecomposition (Eq. 5), then from the eigenvalue spectrum we determine *K*, we continue by constructing the $K-1$-dimensional embedding with the first $K-1$ non-trivial eigenvectors (the minus-1 stems from excluding the trivial eigenvector), and apply *K*-means to the coefficients in this space. This results in *K* clusters.

### Toy example

We illustrate the model and associated clustering in a fictitious transport system consisting of 10 nodes and 14 edges in Fig. 1c,d. Trains go from node to node in a networked manner and their (bidirectional) frequencies are denoted in numbers on each edge, creating geographical differences in $\alpha$. The factor $\beta$ is assumed here to be constant: $\beta = \beta _0=1$. The resulting *M* matrix gives the first non-trivial eigenvector as displayed in colors in the nodes in Fig. 1c. By eye already, one can distinguish the red colored nodes (coefficients $>0$) from the the blue colored nodes (coefficients $<0$), which also makes sense dynamically: even though connections between nodes 4 and 6, and between 2 and 8 exist, they are much weaker than the interconnections between nodes 1–5, and subsequently do not bring the coefficients (i.e., of 4/6 and 2/8) of the first eigenvector close together. It turns out that for this system, $K=3$. The resulting clustering is found in Fig. 1d, confirming our observations by eye on the coefficient separation in Fig. 1c: the algorithm groups the interconnected region of 1–5. It also distinguishes nodes 6 and 7 from 8–10, as a result from the strong connection between 6 and 7. While this is only a fictitious toy network, in the following sections, we apply the same algorithm to real and much larger transport networks.

## Application to European railway systems

We apply the algorithm to data of nation-wide railway systems of four European countries: the Netherlands, Italy, Germany and Switzerland. We have chosen these systems based on their relative comparability: railways in the United States, for example, have a strong emphasis on cargo transport (in contrast to European railways, having more emphasis on passengers) affecting frequency and regularity of the timetables, and even various topological aspects. Another example is the Chinese railway system, differing from European systems in terms of scale: having fewer stations (per unit area) and much longer running times. Hence, for the illustration of the methods in this paper, we focus on four railway systems that are relatively comparable, but still have smaller cross-differences. We start this section by elaborating on the data itself and topological properties of these systems, after which we present the spectral results of the *M* matrix for every of these four systems. We end this chapter with showing the resulting clusters.

### Data

We utilize operational data from the Dutch, Italian, German and Swiss railway systems, including data on infrastructure, schedules (used to determine the values of $\alpha _{ij}$ for every edge *ij*) and realized delay data (used to determine the values of $\beta _{ij}$). Details on the source and cleaning of the data can be found in Supplementary Information A. The data contains departure and arrival times, locations and their delays, per unique train number, along with infrastructure information on stations, their connections and longitude–latitude data. We obtain daily average variables like frequencies and running times by looking at periods of 16 to 31 days (depending on the country, see Supplementary Information A). Frequency is computed as average number of trains per hour by summing the daily amount and dividing by 24. A minimum value of 1 minute running time (in hours) is taken: i.e., lower average running times are approximated to 1 minute. In Table 1, an overview of several static properties of the railway systems is given. Insights in the networks themselves is supported by also showing two network metrics: the average *degree*, which refers to the average amount of links each node has, and the average *betweenness*, which is the fraction of shortest paths between all pairs of nodes that pass through a respective node, averaged over all nodes.

**Table 1:** General overview of the four countries assessed in this paper. Node numbers are determined after removal of nodes that are not connected to the giant component. *Here, we show the average *non-diagonal* $\alpha$ and $\beta$ values.

Variable	Netherlands	Germany	Switzerland	Italy
# connected nodes	658	5815	1346	2201
# edges	1438	15764	3827	6683
Average degree	2.26	2.90	2.56	3.26
Average betweenness	0.048	0.0052	0.0095	0.013
Average daily # train activities per node	173	20.54	102.35	22.47
Average daily # unique service lines per node	10.8	2.2	10.3	2.8
Average $\alpha ^{\text {*}}$	0.201	0.117	0.132	0.067
Average $\beta ^{\text {*}}$	1.009	0.974	0.837	0.991

In Table 1, we can see several differences across the four systems. When interpreting these numbers, it is good to emphasize that the distance between nodes and the level of detail varies across the four data sets: for example, in the Netherlands, the data is more detailed the level of passenger stations: there are sensors near the tracks (also outside of stations) that log whether trains are passing by. This level of resolution is higher for the Dutch case than in the other sets, affecting static properties of the system like reducing average running time between edges or average degree, for example. (This resolution difference will not affect cross-system comparisons in later figures.) But even when taking this note into account, we conclude that the German railway system is clearly the largest, in terms of both nodes and edges. The low average degree and high average betweenness in the Netherlands can be explained by the fact that it includes many degree-2 nodes sequential on a line (by construction, but partly also due to the high resolution of the data), rather than having larger hubs that are interconnected.

**Figure 2:**
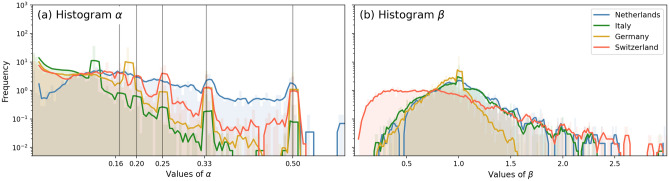
Normalized histograms of occurrence of $\alpha$ and $\beta$ values in the four analyzed countries, excluding diagonal elements of the $\alpha$ and $\beta$ matrices. In panel (**a**), the vertical lines indicate fractions of 1, marking $\alpha$ values that are associated with proportional train frequencies or integer-minute running times.

From a dynamical perspective, the values about service lines and train activities are more relevant. Service lines are defined as single-direction trips from a starting station to an ending station (commonly at the other side of the country), crossing various in-between stations. These lines are denoted with a unique number and are important advectors of delay^14^, which is why their structure is important for railway dynamics. The smaller systems (Switzerland and the Netherlands) clearly dominate with respectively 10.3 and 10.8 unique lines per node, in comparison to Germany and Italy, having values of 2.2 and 2.8, respectively. This reflects the dynamically denser nature of the Dutch and Swiss railway systems, and is confirmed in the higher number of daily train activities per node (102–173 for the Netherlands and Switzerland as opposed to 20–22 for Italy and Germany).

The average $\alpha$ and $\beta$ values also have an important meaning. They are proxies for the density of trains—modulated by frequency and running time—and statistical delay increases (on edges), respectively. A relatively high $\alpha$ in the Netherlands and, to lesser extent, Switzerland depicts relatively high train density between stations and tracks of degree-2 nodes to prevent scheduled fragmentation of delay spread. The $\beta$ values are like a railway edge-performance metric: the lower, the less delay is increased along its edges. Interestingly, the dense, highly utilized Swiss railway network performs best when looking at these numbers, having an average $\beta$ of 0.84. (Note that for a full performance comparison among these railway systems, one should statistically correct for a number of factors like delay changes *within nodes*, which is not included here.) The histograms of $\alpha$ and $\beta$ of the four countries are shown in Fig. 2a,b, respectively. In several countries, $\alpha$ values correspond to integer-fractions of one: 1/2, 1/3, 1/4, etc., reflecting integer-minute running times on tracks where only 1 line is traveling, or the respective proportions as calculated from the relative train frequencies (see Fig. 1a). This is most notable in Switzerland and the Netherlands, where we expect such short tracks (of 1, 2, 3, 4 or 5 minutes running time) and many degree-2 nodes to exist, because of the high density of these railway systems. The Italian system has more lower values of $\alpha$ than the other countries (with the Dutch system having the fewest), corresponding to either tracks connected to nodes with trains in many (other) directions, or tracks with large average running times. In panel (b), the distributions of $\beta$ are wrapped around 1, corresponding to no significant average change in delay on these tracks. Furthermore, we see that Switzerland has more $\beta$ values lower than 1 in comparison to other countries—the latter two mainly having values of $\beta$ close to 1.

### Spectral results

**Figure 3:**
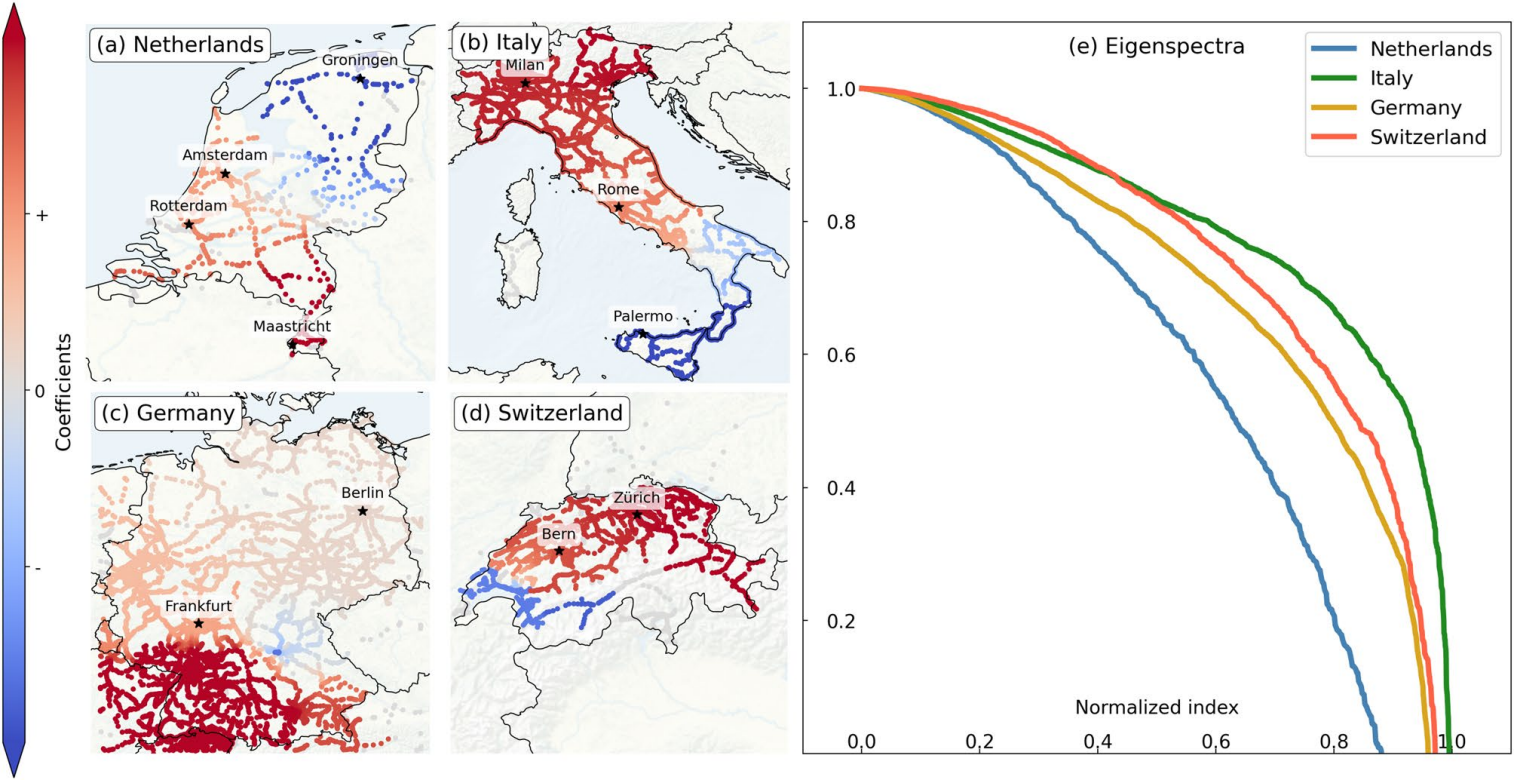
Panel (**a**)–(**d**) First non-trivial eigenvectors of the *M* matrix, for the four European railway systems: (**a**) the Netherlands, (**b**) Italy, (**c**) Germany and (**d**) Switzerland. As absolute values of the coefficients are not relevant (only their relative magnitude to other ones in the same country are relevant), the colorbar and coefficients are shown normalized. Several important cities are annotated by black stars. Panel (**e**) Eigenspectra of the four countries, with normalized horizontal axis. Maps were generated using the Cartopy package in Python.

From the values of $\alpha$ and $\beta$, we determine the matrix *M* using Eq. (3). Figure 3 shows the first non-trivial (i.e., second) eigenvectors of the four countries in panels (a)–(d), and the associated eigenspectra in panel (e) (with a normalized horizontal scale).

The coefficients of the first non-trivial eigenvector in all four countries show a dipole-like structure. In the Netherlands (Fig. 3a), the coefficients depict a north-south gradient, highlighting a northern region (near Groningen) in negative coefficients and the south (towards Maastricht) in positive coefficients, with a better connected center in between, including cities like Rotterdam and Amsterdam. The Italian coefficients (Fig. 3b) also show a north-south gradient. Germany (Fig. 3c) shows a clear separation of the area south of Frankfurt (including cities like Karlsruhe and Stuttgart) in red. Apparently, this area may have persistent delays that are less easily exchanged with the rest of the country. The Swiss graph (Fig. 3d) shows, like in the Dutch and Italian case, a geographic gradient: from east to west, highlighting the south including the cities of Geneva, Lausanne and Sion on the west.

Figure 3d shows the eigenspectra of the four countries, with a normalized horizontal axis to compare the spectra independent of network size. The eigenvalues are relatively high. The slow decrease of the eigenspectra indicates that these are not strongly disconnected clusters. The relatively faster decrease of the Dutch eigenspectrum reveals that in the weighted network spun by *M*, the delay changes in Dutch railway system is more strongly dominated by the first few components, as opposed to others. This reflects that the first *K* eigenvector are better suited for compartmentalizing the Dutch railways into subregions, than they are for other countries.

The (simple) dipolic structure of these eigenvectors and the fact that there are many high-value eigenvalues in the spectrum both point to the need of multiple eigenvectors to obtain a more refined view of dynamic structures in these networks.

### Clustering results

**Figure 4:**
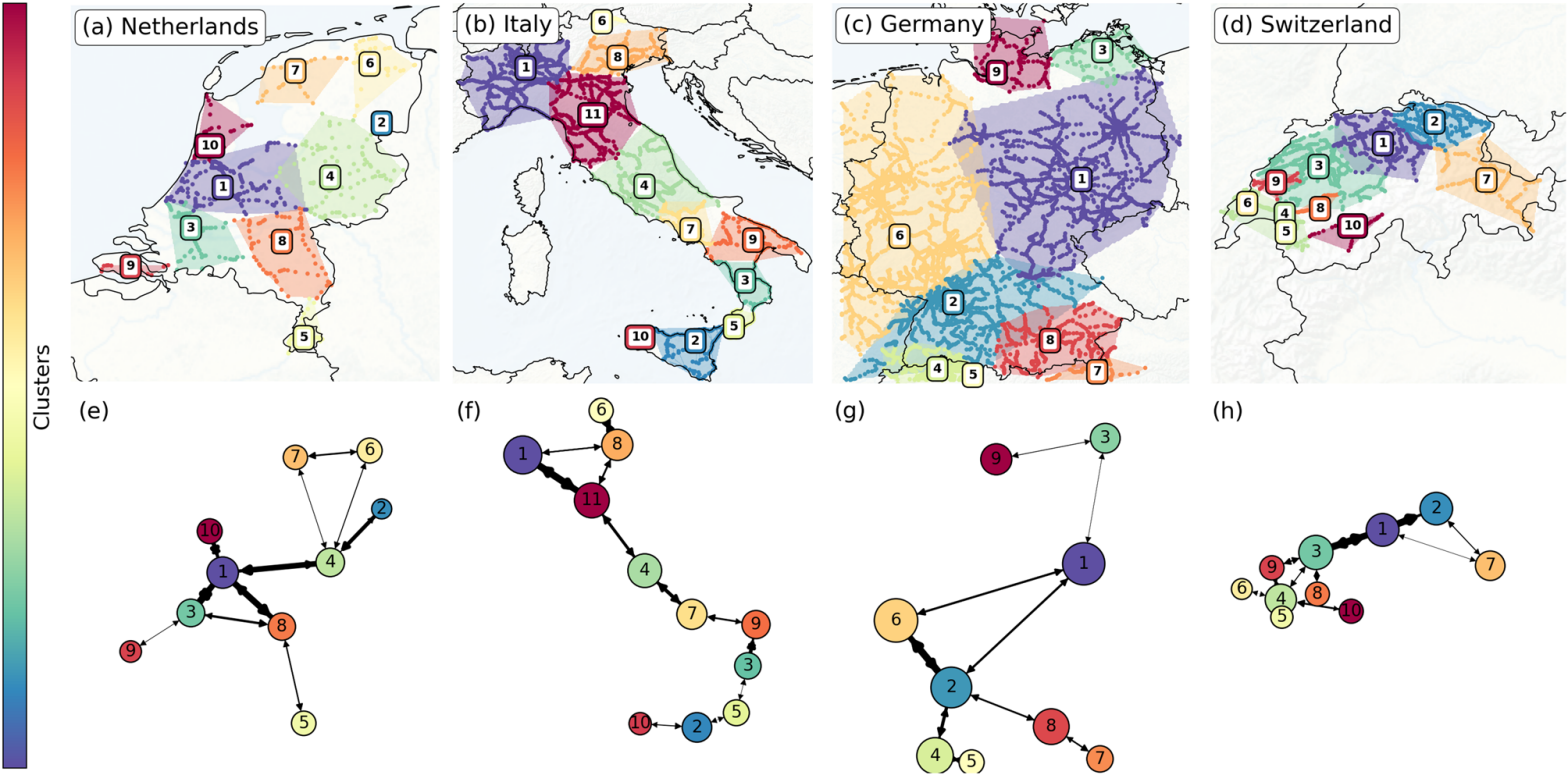
Panel (**a**)–(**d**) Clusters in the four European railway systems as found using *K*-means on the embedding built by the first $K-1$ non-trivial eigenvectors of the matrix *M* (where *K* is found using the eigengap heuristic). In particular, *K* equals respectively 10, 11, 9 and 10 for these four countries. Panel (**e**)–(**h**) Abstracted versions of upper panels, reflecting the clusters and their connections. Arrows indicate connectivity between clusters in terms of $\alpha$ and $\beta$—the elements of *M*. The arrow width depicts the total sum of elements of *M* directed from one cluster to another. Node sizes reflect amount of railway stations in each cluster. Maps were generated using the Cartopy package in Python.

Using the eigengap heuristic and the eigenspectra in Fig. 3d, we determine *K*—the number of clusters to search for, and the dimension of the embedding (see “Clustering”). Subsequently, we apply the *K*-means clustering algorithm to the eigenspace and find the clusters shown in Fig. 4, with the geographic locations of the clusters in panels (a)–(d) and abstracted networks (including delay exchange in the arrow widths) in panels (e)–(h).

In general, a total of 10, 11, 9 and 10 clusters are found in the railway systems of the Netherlands, Italy, Germany and Switzerland, respectively. By nature of the planar (2D-) structure of railway networks, the clusters are geographical intraconnected regions. In the Netherlands (Fig. 4a), the country is cut up in mostly equal-sized regions, with exceptions of smaller clusters (2, 5, 9) at the edges of the country (e.g. in the rural areas of Zeeland in the south-west, Limburg in the south, and a poorly connected part in the north-east)—probably as a result of service lines being less frequent and more disconnected from the center of the country in these areas. Easily spotted are the central cluster 1, including the major cities of Amsterdam and Utrecht, and cluster 3, including Rotterdam, together including most of the so-called ‘Randstad’, the most urbanised and transport-heavy area of the Netherlands. The clustering in Italy (Fig. 4b) also subdivides the country into more-or-less equal areas, with some exceptions of small clusters in the far north and south (clusters 5, 6, 10). Three notable clusters can be identified, including important Italian cities: 1, being the largest with Milan and Turin, 11, including most of Tuscany and Bologna, and 4, including Rome. The island of Sicily is only weakly connected to the rest via a train ferry at the city of Messina, and the island is further subdivided into two other clusters. Germany (Fig. 4c), in contrast, is subdivided in only 9 clusters (although being the largest railway system among these four) that are not equal in size: clusters 1, 2, 6 and 8 add up to than 85% of the country, including several foreign tracks. Cluster 6 almost perfectly coincides with the German federal states (‘Bundesländer’) of Nordrhein-Westfalen, Hessen and Rheinland-Pfalz, while cluster 1 covers most central-eastern states (these Bundeslände are also used in operations by the dominant railway company, Deutsche Bahn). Several much smaller clusters can be found in the south: clusters 4, 5 and 7, even being foreign (non-German) clusters and hence not of importance here. The large size of the clusters reflect that railway transport on the tracks are generally well distributed, travel long distances, and are less regional. The Swiss partitioning (Fig. 4d) results in rather small, intricately connected clusters (as is also visible in the abstracted graph in Fig. 4h). Several major urban areas can be recognized like the Geneva-Lausanne area (cluster 4) and the area around Bern (cluster 4), while the area around Zürich is divided into two clusters (cluster 1 and 2).

In the abstracted plots [panels (e)–(h)], the arrows and their widths provide information on the general flow of delay, proxied by the elements of *M*: thick arrows mean more (and larger) elements of *M* between these clusters, indicating stronger pathways of delay propagation as dictated by the $\alpha$ and $\beta$ values, while small arrows mean the opposite. The Netherlands has a relatively well connected dynamical core in its center: exchanging most delay among the four clusters 1, 3, 4 and 8, containing several major transport corridors for both passengers and freight. The Italian plot indicates that most exchange is in the center and north, between clusters 1, 8, 11, 4 and 7, reflecting touristic and urbanized areas, possibly leading to well-connected railway operations. The German clusters are mostly connected in the west and south: forming a strong bond between 2 and 6 in particular, reflecting some international transport and core lines from Cologne and Frankfurt to Stuttgart. The core connections of the clusters in Switzerland are mostly along the northern border, connecting the cities of Bern and Zürich.

## Interpretation and relation to daily operations

The clusters reflect regions where delay is expected to to be propagated within the cluster, and less so towards outside of the cluster, incorporating the two factors of spatial non-uniformity. This section is devoted to interpret and understand the relevance of these clusters in terms of the delay and daily operations. We start by defining two metrics that allow such interpretation and apply them to the clusters found in Fig. 4. Subsequently, we compare the four countries and their clusters in terms of these metrics.

### Metrics to interpret clusters

To find the operational and dynamical meaning of the clusters found in Fig. 4, we first define a few relevant quantities that we later combine into two cluster-characterizing metrics. We attribute any train’s delay to its *departure* location. The first quantity is the total delay $D_{\text {total}}$ from stations in the cluster. Second, we determine the internal delays $D_{\text {int}}$: delays of trains departing and arriving within the cluster. Third, exported delays $D_{\text {exp}}$ of trains departing from within the cluster, but arriving in another, are computed. And fourth, the imported delays $D_{\text {imp}}$ of trains departing from another cluster, arriving inside the analyzed cluster. The following relation holds: $D_{\text {total}} = D_{\text {int}}+D_{\text {exp}}$. With these quantities, we compute two metrics which allow for easy interpretation of the clusters: (1) their fraction of the country-wide delays, measured by the *cluster severity* and (2) their dynamical (dis-)connectedness to other clusters, proxied by the *cluster independence*.

Cluster severity *S*(*n*) for any cluster *n* is the ratio of $D_{\text {total}}$ of cluster *n* to the average $D_{\text {total}}$ over all clusters:

7 $$\begin{aligned} S(n) = \frac{D_{total}(n)}{\frac{1}{N}\sum _i^N D_{total}(i)} \end{aligned}$$

with *N* being the total amount of clusters. So, if $S(n) >1$, the cluster covers an above-average part of the delays in the country, and vice versa. This does not incorporate cluster size, meaning that if all stations cover equal amounts of delay, larger clusters immediately have larger values of *S*(*n*). We have chosen to not account for cluster size to make *S*(*n*) a property of the cluster as a whole, make it better interpretable and relate to the practical use of the metric: small clusters might otherwise attain very high values of *S*(*n*) while in practice not that dominant in delay.

Cluster independence *I*(*n*) for any cluster *n* is defined as the ratio between delays exchanged internally in the cluster, w.r.t. the delays exchanged with other clusters:

8 $$\begin{aligned} I(n) = \frac{D_{int}(n)}{\sqrt{G(n)}\cdot (D_{imp}(n)+D_{exp}(n))}, \end{aligned}$$

where *G*(*n*) is the number of nodes in cluster *n*. The factor $\frac{1}{\sqrt{G(n)}}$ is included to counteract the bias that larger clusters automatically have more internal delays ($D_{int}(n)$) than they exchange with neighboring clusters ($D_{imp}(n)+D_{exp}(n)$). We assume here that the total internal delay grows with the amount of stations in the cluster (e.g., $D_{int}(n)\propto G(n)$) and that the delay exchange grows with the square root of that (e.g., $D_{int}(n)\propto \sqrt{G(n)}$)—much like the area of a circle grows with the radius squared, but the circumference merely grows with the radius.

Note that we do account for the cluster size bias in *I*(*n*), but not in *S*(*n*). This might seem inconsistent. However, *S*(*n*) is already normalized by the average value of total delays in the clusters, while *I*(*n*) is not a normalized value. The quotient of internal delays versus delay exchange (i.e., $I(n)\cdot \sqrt{G(n)}$) will vary greatly and therefore, adding $\sqrt{G(n)}$ as a normalization factor benefits the interpretation of *I*(*n*) to be this quotient relative to the cluster size. More specifically, the resulting interpretation would be that $I(n) =1$ for all clusters where the internal delays are exactly $\sqrt{G(n)}$ as large as the delay exchange, allowing a cross-comparison of large and smaller delays. Note that we proxy (and refer to) dynamical ‘connectedness’ with *I*(*n*), which formally only measures the amount of delay exchange with surrounding clusters. In the text, this terminology is interchangeably used.

The metrics *S*(*n*) and *I*(*n*) both surround values of 1: values lower than 1 depict clusters that cover a less-than-average delays and are well-connected, respectively, and values higher than 1 reflect clusters with more-than-average delays and that are less connected. This allows to split the $I(n)-S(n)$ plane into four parts, that can be used to interpret the clusters. Four cluster categories can be distinguished, as displayed in Table 2: named for easier reference Type A, Type B, Type C and Type D clusters.

**Table 2:** Cluster categories in the $I(n)-S(n)$ plane.

	$S(n)<1$	$S(n)>1$
$I(n)>1$	**Type A**	**Type B**
	Less delay exchanged with other clusters	Less delay exchanged with other clusters
	Small amounts of originating delay	Large amounts of originating delay
$I(n)<1$	**Type C**	**Type D**
	More delay exchanged with other clusters	More delay exchanged with other clusters
	Small amounts of originating delay	Large amounts of originating delay

### Metric results

**Figure 5:**
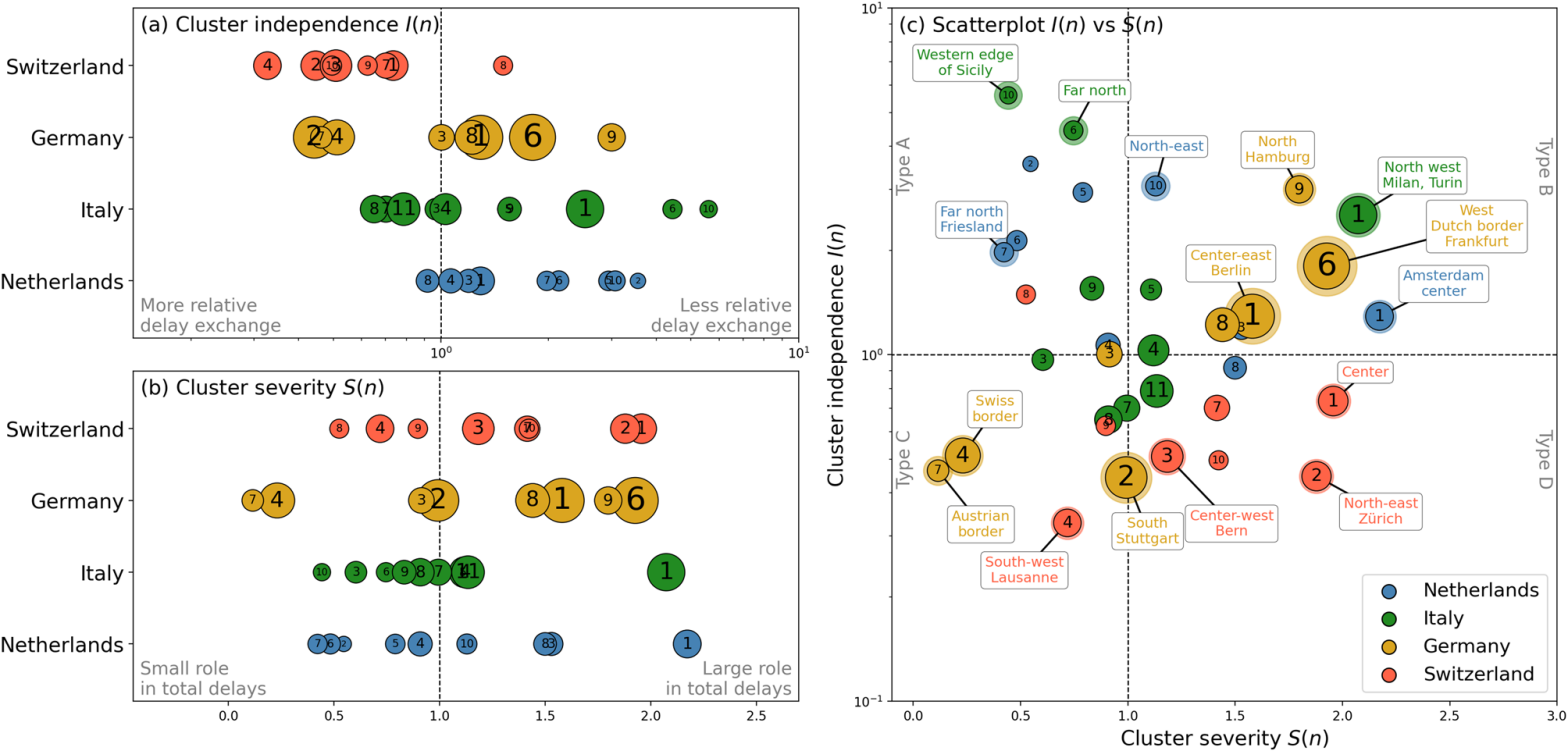
Cluster independence *I*(*n*) and cluster severity *S*(*n*) of the clusters across the four analyzed countries. Panels (**a**)–(**b**) *I*(*n*) and *S*(*n*), respectively, on the horizontal and countries on the vertical. Panel (**c**) Scatterplot with *I*(*n*) versus *S*(*n*). Several notable clusters are annotated in text and their dots are emphasized. Dot size reflects the number of stations inside the cluster. Clusters with only a very small amount of data (e.g. due to being across the border, involving only international trains) are not included in this figure. Numbers in the clusters refer to numbers in Fig. 4.

The values of *I*(*n*) and *S*(*n*) for all clusters *n* across the four countries (in colors) are plotted in Fig. 5 (a few clusters with too little data to estimate *I*(*n*) and *S*(*n*) well are left out). The numbers in each circle refer to the numbers in Fig. 4.

The panels (a) and (b) allow for both an in-country comparison of the clusters as well as a cross-country comparison of the spread of the *I*(*n*) and *S*(*n*) values. In panel (a), the immediate observation is the relatively low *I*(*n*) values in Switzerland in contrast to relatively high values in the Netherlands, with Germany and Italy approximately in between. Apparently, many clusters in the Swiss system exchange more delays with other clusters than they have internally. This may be a consequence of the fact that this relatively small country has many trains connecting clusters, rather than traveling in a separated space. This points to a potential source of vulnerability: delay can quickly spread across the country. Looking more closely to the cluster numbers within each country, we can find mostly urban and central areas having low *I*(*n*) values and regions place at the edges of the country having higher *I*(*n*) values. Examples are the area of Zürich, Bern and Lausanne (numbers 2, 3, 4) having low-*I*(*n*) in Switzerland, while the Milan region (1) and several regions at the edges (Sicily and far north) in Italy have much higher *I*(*n*) values. In the Netherlands, the highest *I*(*n*) values are found in rural areas in the north and south. An exception seems to be Germany, where clusters 1 and 6 are so large that they convey well-separated, but urban areas.

The *S*(*n*) values in panel (b) are by definition spread around the value of $S(n)=1$, because *S*(*n*) is normalized by its average (Eq. 7). Still, the spread provides valuable information on these countries. While the Swiss clusters are relatively well spread across the average, the German systems have a few clear outliers on both sides: the southern border (4 and 7) clearly having the smallest fraction of delays, whereas the area around Köln (6) has the largest fraction of delays. Both Italy and the Netherlands show many clusters in the low-*S*(*n*) domain, with a few outliers with high *S*(*n*) values, mostly in the bigger cities—Amsterdam, Utrecht and Rotterdam (1 and 3) in the Netherlands, and Milan (1) in Italy.

In panel (c), the clusters are shown in the combined $I(n)-S(n)$ plane. The top-left quadrant (Type A clusters) mainly comprises Dutch and Italian rural clusters, such as the far north or south in these countries: the areas of Friesland and Limburg in the Netherlands and Sicilian and northern areas in Italy. The rural nature of clusters in this category is no surprise, as their periphery-located position in the network make them usually less connected with the rest (i.e., high *I*(*n*)) and less prone to delays (i.e., low *S*(*n*)). The Type B clusters ($I(n), S(n)>1$) are mainly found in Germany, with the busy, well-connected areas around Berlin, Köln and Hamburg. One other notable cluster is the urban Milan region in the north of Italy (cluster 6), clearly separated from other Italian clusters. At the bottom-left we see the Type C clusters, containing several German, Swiss and Italian clusters that are quite well connected, but play less of a role in the total delay. The Type D clusters (bottom-right), are mainly found in Switzerland, interpreted as well-connected clusters that also play a large role in the total delay. The Swiss Type D clusters are the relatively urban regions in this country around Zürich and Bern.

Summarized, comparing countries in the $I(n)-S(n)$ plane reveals that Switzerland generally has low values of *I*(*n*), implicating the strong connectivity of this railway network. Germany is not really represented in the Type A and Type D categories, and is well represented in Type B. This may reflect that German delays are usually quite compartmental: large delays may arise but these large clusters mainly keep these delays within. The Italian and Dutch clusters show a large spread for values $S(n)<1$, and several clusters around $I(n)=1, S(n)>1$. There seem to be a few clusters in both of these countries that determine a disproportionally large amount of the total delay (high *S*(*n*)), but these clusters have an average connection with the rest of the country (unlike several German clusters). Overall, the patterns in Fig. 5 seem to relate to operational characteristics, topological embedding in the general railway network and even urban differences across the clusters.

## Conclusion

To find geographical delay structures that have a dynamical meaning in railway systems, we developed a graph-based model and proposed a method to use spectral properties of this model to characterize the railway systems’ delay dynamics geographically.

In particular, combining spatial non-uniformity factors that can be derived from the timetable and from statistics, all model information is encapsulated in the dynamical matrix *M* whose spectral properties can be used to compartmentalize any transport system’s geographical network into clusters. We analyze four European nation-wide railway systems: the Netherlands, Italy, Germany and Switzerland. Both infrastructural and operational data are used, revealing dynamic properties of the four countries (Table 1)—making an interesting comparison in itself—and an optimal partition. The clusters vary in size and are connected in different ways to the nation-wide networks, resulting in the identification of core, central clusters and peripheric, near-disconnected clusters. The operational meaning of the clusters is expressed in two variables: cluster independence *I*(*n*), reflecting the dynamical (dis-)connectedness of the cluster to the rest of the country, and cluster severity *S*(*n*), reflecting the fraction of delay the cluster is responsible for. This leads to the classification of four cluster types, showing that high-*S*(*n*) values are usually obtained by the more urban and dense clusters (e.g., high values relating to busy areas like the region around Milan, Zürich, Frankfurt and Amsterdam), and that *I*(*n*) is partially distinguishes rural regions from central regions, but is partially also affected by how a country is operationally handled.

Throughout the paper, various comparisons have been made between these four countries, revealing the Netherlands and Switzerland to be dynamically dense railway networks (i.e., having high utilization of their tracks), while Germany and Italy are much larger and sparser networks. Shorter steps between (logging) stations make the edges on average small in the Netherlands, with the longest edges in Italy. The clusters in Switzerland show low values of *I*(*n*), depicting a strong interconnectedness among them. The Italian *S*(*n*) is dominated by the Milan region, with almost all other regions having $S(n)<1$. The upper-right corner of the *I*(*n*), *S*(*n*) plane is dominated by several German clusters near Hamburg, Berlin and Köln.

The matrix *M* consists of quantities $\alpha$ and $\beta$ that are based on average quantities: single values per edge of the railway networks. It should be noted that, in reality, many ingredients of these quantities, such as running times, train frequency (in Eq. 1) and the delay statistics in Eq. (2) may vary significantly over time and per train. Also the spatial non-uniformity factor $\beta$ derived from delay statistics is dependent on seasonality and weather. Working with average quantities limits the potential of simulating specific instances of delays. Additionally, the model does not incorporate node-specific aspects built in the timetable, such as dwell time supplements and buffer times—the model rather focuses on spatial non-uniformity on the edges to assess the directions and speed of delay propagation. These limitations assert that this model should not primarily be used for prediction purposes, but rather to gain time-average and system-wide understanding of geographic delay evolution.

Railways, by schedule, have long-stretched service lines going from one side of the country to the other. Per definition, this means that perturbations quickly pass through large parts of the country, as is also reflected in the high eigenvalues of *M* (Fig. 3). The structure of the dynamics therefore does not favor partition much, and care should be taken into separating them too strongly in the interpretation of Figs. 4 and 5. Still, compartmentalization is an important topic in railways, in particular when it comes to defining regions to subdivide operations and dispatching. The proposed clustering should therefore be viewed more as a statistical average, or a composite of dynamic modes, rather than a full geographical segregation of delays. For future research, the connection and strength between the found clusters should be compared with delay propagation on a larger spatial scale: the relation between regional effects and nation-wide effects is still an important unresolved topic in transport literature. This work brings us one step closer to a solution.

For both scholars and railway practitioners, these results shape deeper understanding of how their railway systems work, how they differ from each other, and how regions with these systems each play a unique role. While the country-by-country statistics in Fig. 2 and Table 1 add to system-wide insights, the clustering in Fig. 4 may help practitioners to find boundaries when aiming to subdivide countries into smaller operational regions. Connections among the clusters may inform railway operators of statistically average directions of delay flows. And the characterization of the clusters by *I*(*n*) and *S*(*n*) in Fig. 5 provides insights in how analogies between countries and regions can and cannot be made, and how different regions play different roles. For scholars, the construction and subsequent clustering of the *M* matrix is a simple procedure and can be generalized to many other transport systems, beyond railways alone. While delay is a quantity strictly bound towards a predefined transport schedule, in theory many other dynamic variables on networks can be analyzed in the same manner. We believe that the presented methodology and results for the European countries contributes to deeper understanding of these systems, and we hope that the paper ignites more research in the relation between regional effects and nation-wide effects in transport systems.

## Supplementary Information

Supplementary Information.

## Data Availability

The analyses are done on publicly available data. For more information, see Supplementary Information A.
