# Supplementary material for: Geographic delay characterization of railway systems
Source: Sci Rep. 2021 Oct 21;11:20860. doi: 10.1038/s41598-021-00361-z (PMC8531349; doi:10.1038/s41598-021-00361-z)
Supplement: Supplementary file 1 — Supplementary Information. [file 41598_2021_361_MOESM1_ESM.pdf]

Geographic delay characterization of railway systems
Supplementary Materials

Mark M. Dekker[1,2,*]

[1]Department of Information and Computing Sciences, Utrecht University, Princetonplein 5, 3584 CC Utrecht, The Netherlands
[2]Centre for Complex Systems Studies, Utrecht University, Minnaertgebouw, Leuvenlaan 4, 3584 CE Utrecht, The Netherlands
[*]m.m.dekker@uu.nl

A Data sources and processing

A.1 Data sources

Operational data for the Netherlands is obtained from the Dutch infrastructure manager ProRail and covers the period July 2018 to June 2019. A selection of 16 days is made, based on delay severity (4 days in each of four categories; Green, Neutral, Red and Black, for more details see the Appendix or[1] and[2]), taken to statistically cover multiple situations. Network data on locations and links of the nodes and edges of the railway network, including delay time series can be found in an Open Science Framework related to previous work[2]: `https://osf.io/tps4r/`. Data for the German and Italian railways are obtained from the supplementary material of[3], who received this data from the OpenDataCity (`http://www.opendatacity.de/`) and the ViaggiaTreno (`http://www.viaggiatreno.it/`) websites. The German data spans across March 2015, and the Italian data across March 2015 and April 2015. The Swiss railway data is obtained from the OpenTransportData website (`https://opentransportdata.swiss/`) over January 2018. The German data consists of several stations across the border in mainly the south and west, which are strongly connected to its lines.

A.2 Data cleaning

From the infrastructure data, the network topologies of these systems is determined. In all but the Dutch case, the data had to be cleaned for unrealistically long edges. These edges arise from trains traveling long distances between major cities without stopping in subsequent smaller stations, effectively adding direct edges between major cities that in practice are not direct edges. This cleaning is done by looking at the distribution of edge lengths. Also, because the timetable may vary from day-to-day, I set the minimum train frequency on any to be 1 per hour, and scheduled running times to be at least 1 minute, to avoid values of α to become zero or infinity.

Furthermore, it is important to note that there are disconnected (e.g. Sardinia in the Italian case) and non-operational components in some of the railway systems analyzed in this paper. These parts are omitted - we focus on the giant component and where necessary, make all links bidirectional. Note that these problems only concern a small fraction of the links in these systems.

A.3 Train types

We focus on passenger trains only, and exclude freight trains in the analysis. One reason for this is that freight trains are (economically) privacy sensitive, meaning that it is difficult to get a complete dataset. Another reason is that delay is not always well-defined for such trains, as their routes and schedules are mostly separated from the main schedule. It should be noted that their contribution to delay is usually only minor, as, for example in the Netherlands, they cover only 5.7% of all Dutch train kilometers in 2017 (numbers courtesy of the Dutch infrastructure manager ProRail). More details can be found in Dekker et al. (2019)[2], section 3.1.

References

1. Dekker, M. M. & Panja, D. Cascading dominates large-scale disruptions in transport over complex networks. *PLOS ONE* **16**, 1–17, DOI: 10.1371/journal.pone.0246077 (2021).

2. Dekker, M. M., Panja, D., Dijkstra, H. A. & Dekker, S. C. Predicting transitions across macroscopic states for railway systems. *PLOS ONE* **14**, e0217710, DOI: 10.1371/journal.pone.0217710 (2019).

3. Monechi, B., Gravino, P., Di Clemente, R. & Servedio, V. D. Complex delay dynamics on railway networks from universal laws to realistic modelling. *EPJ Data Sci.* **7**, 35, DOI: 10.1140/epjds/s13688-018-0160-x (2018). 1707.08632.
